# Clinical characteristics and prognosis of patient with leptospirosis: A multicenter retrospective analysis in south of China

**DOI:** 10.3389/fcimb.2022.1014530

**Published:** 2022-10-17

**Authors:** Dianwu Li, Huaying Liang, Rong Yi, Qian Xiao, Yiqun Zhu, Qinyu Chang, Lihua Zhou, Bin Liu, Junjun He, Tianxing Liu, Zhijun Fan, Wei Cheng, Weizhong Wang, Yan Zhang, Pinhua Pan

**Affiliations:** ^1^ Department of Respiratory Medicine, National Key Clinical Specialty, Branch of National Clinical Research Center for Respiratory Disease, Xiangya Hospital, Central South University, Changsha, China; ^2^ Center of Respiratory Medicine, Xiangya Hospital of Central South University, Changsha, China; ^3^ Clinical Research Center for Respiratory Diseases in Hunan Province, Changsha, China; ^4^ Hunan Engineering Research Center for Intelligent Diagnosis and Treatment of Respiratory Disease, Changsha, China; ^5^ National Clinical Research Center for Geriatric Disorders, Xiangya Hospital, Changsha, China; ^6^ Department of Pulmonary and Critical Care Medicine, Zhuzhou Central Hospital, Zhuzhou, China; ^7^ Department of Anaesthesiology, Hunan Provincial People’s Hospital, Changsha, China; ^8^ Department of Respiratory Medicine, Changsha Central Hospital, Changsha, China; ^9^ Department of Emergency, Xiangtan Central Hospital, Xiangtan, China; ^10^ Department of General Surgery, Shaoyang Central Hospital, Shaoyang, China; ^11^ Department of Orthopaedic Surgery, Yongzhou Central Hospital, Yongzhou, China; ^12^ Department of Cardiothoracic Surgery, Liuyang People’s Hospital, Liuyang, China; ^13^ Department of Respiratory, The Second Xiangya Hospital of Central South University, Changsha, China; ^14^ Department of Respiratory, The First Affiliated Hospital of University of South China, Hengyang, China

**Keywords:** leptospirosis, mortality rate, prognosis, temperate zone, China

## Abstract

**Purpose:**

Leptospirosis is a zoonotic disease caused by pathogenic spirochetes of the genus Leptospira. However, there is currently no consensual definition or diagnostic criteria for severe and different forms of leptospirosis. Therefore, more insight on clinical manifestations, risk factors, and outcomes of leptospirosis is warranted. The identification of leptospirosis with distinct clinical manifestations and prognosis in our population.

**Methods:**

Multiple correspondence analysis and hierarchical classification on principal components were presented to identify different clinical types of leptospirosis. The outcomes were clinical phenotypes, laboratory and imaging findings, and prognosis.

**Results:**

The 95 enrolled patients had median values of 54.0 years (39.0-65.0) for age, 9.0 (7.0-14.0) for total hospital stay lengths, of whom 86.3% was male and 40.0% was transferred to ICU. Three clinical types were distinguished: mild leptospirosis (n=43, 45.3%) with less organ dysfunction and shorter hospital stays; respiratory leptospirosis (n=28, 29.5%) with hemoptysis, and respiratory and circulatory failure; and hepato-renal leptospirosis (n=24, 25.3%) with worst liver and kidney dysfunction. Total hospital mortality was 15.8% and was associated with dyspnea and high levels of neutrophil counts.

**Conclusions:**

The identification of leptospirosis with distinct clinical manifestations and prognosis in our population may assist clinicians to distinguish leptospirosis-like disease. Moreover, dyspnea and neutrophil count were found to be independent risk factors for severe leptospirosis progression.

## Introduction

Leptospirosis is a zoonotic disease caused by pathogenic spirochetes of the genus Leptospira, and is one of widespread acute febrile illness throughout the world, with fatality rate ranging as high as 20-25% in some regions (Leptospirosis worldwide, 1999). Leptospirosis is mostly transmitted to humans by ambient water contaminated with the urine of wild and domestic mammals that have been chronically colonized by Leptospira (Bharti et al., 2003). Specific occupational activities (e.g., farming, veterinary medicine, military training), recreational immersion in water, poor living conditions, and seasonal rainfall in the tropics are commonly connected with the disease (Bharti et al., 2003). In general, human leptospirosis is prevalent in east/southeast Asia, as well as South America (Ricaldi et al., 2013). However, in temperate zones, such as Europe, North America, and Africa (Hartskeerl et al., 2011; Biggs et al., 2013; Dupouey et al., 2014), it has been reported that the incidence of leptospirosis is increasing, due to global warming, hurricanes, or heavy rains with floods and proliferation of urban rodents (Levett, 2001; Pijnacker et al., 2016).

Even though leptospirosis is a serious but underappreciated infectious tropical illness, it continues to be a major public health concern throughout the world. In recent decades, there is a trend towards increasing incidence in many countries (Lee et al., 2016; Holla et al., 2018; Sasmal et al., 2019) including India, South Korea and America. In some endemic areas (Zhang et al., 2012) of China, there are periodically small-scale local outbreaks of human leptospirosis. Leptospirosis presents a diverse array of clinical manifestations, including jaundice, renal failure, thrombocytopenia, and possibly lethal pulmonary hemorrhage (Levett, 2001). Meanwhile, leptospirosis shares common clinical signs with many acute febrile diseases, such as influenza, dengue fever or malaria (Tubiana et al., 2013), causing various degrees of infections (Doudier et al., 2006). Additionally, some clinical symptoms of leptospirosis have been confirmed to be closely related to death, including oliguria, shock, altered mental status, dyspnea, pulmonary infiltrates, and more (Ko et al., 1999; Doudier et al., 2006; Wang et al., 2020). However, there is currently no consensual definition or diagnostic criteria for severe and different forms of leptospirosis. Leptospirosis has been classified in a variety of methods, ranging from clinical manifestations to genetic (Marquez et al., 2017) determination. Therefore, more insight on clinical manifestations, risk factors, and outcomes of leptospirosis is warranted.

This retrospective multicenter analysis was conducted to determine the association between clinical phenotypes of leptospirosis, laboratory findings, treatment strategies and prognosis.

## Methods

### Patients

We retrospectively extracted and analyzed the clinical profile data on leptospirosis-diagnosed patients who were admitted to 9 tertiary care hospitals in Hunan, China, between January 1, 1998, and January 1, 2022. This study was approved by the institutional review boards (IRBs) in Xiangya Hospital of Central South University (No. 202104005).Leptospirosis was diagnosed as one of the following positive tests: microscopic agglutination test (MAT), enzyme-linked immunosorbent assay (ELISA), polymerase chain reaction (PCR) or metagenomic next-generation sequencing on urine or blood, and dark field microscopy (Bleeker-Rovers et al., 2007; Koizumi, 2020). Clinical profiles including demographics, comorbidities, clinical symptoms, laboratory and imaging findings, and prognosis were obtained from the electronical record.

### Definitions

Patients were classified as having severe leptospirosis (Miailhe et al., 2019) if they had required ICU admission. Acute respiratory failure (Roussos and Koutsoukou, 2003) was defined as a significant impairment of pulmonary gas exchange functions with inadequate oxygenation (PaO2 <60 mmHg) and carbon dioxide retention (PaCO2 >45 mmHg). Circulatory failure was classified as a collection of clinical syndromes caused by cardiac dysfunction that were characterized by pulmonary congestion, systemic congestion or tissue hypoperfusion. Acute liver injury was characterized as liver damage induced by a variety of factors in the absence of a history of chronic liver disease, accompanied by an increase in serum transaminase and bilirubin levels. Acute renal failure (Ronco et al., 2019) was defined as at least one positive laboratory test within 7 days of the following: creatinine greater than 1.5 times baseline (or a rise of 0.3 mg/dL within any 48-hour period), or urine output less than 0.5 mL/kg for more than 6 hours. Multiple organ failure was described as the dysfunction of two or more organs following severe injury.

### Statistical analysis

All data in the current study was analyzed with SPSS 26.0 software (IBM SPSS Inc., USA). Continuous variables with skewed distribution were presented as medians and interquartile ranges, and compared by the Kruskal-Wallis H test. Categorical variables were expressed as frequencies and percentages (%), and analyzed by Chi-square test or Fisher’ s exact test. In addition, risk factors were assessed univariately, and variables of statistical and clinical significance in univariate analysis were including in the multivariate logistic regression analysis.

In order to identify different clinical phenotypes of leptospirosis, multiple correspondence analysis (MCA) and hierarchical classification on principal components (HCPC) were conducted using R 4.1.3 (FactoMineR package). The variables including age, sex and smoking history; clinical symptoms on admission (fever; fatigue; myalgia of the lower limbs; arthralgia; altered consciousness; headache; jaundice; vomiting; diarrhea; abdominal pain; dyspnea; cough; hemoptysis; other bleeding such as gastrointestinal haemorrhage, epistaxis, purpura and haematuria); organ dysfunction (acute respiratory failure; circulatory failure; acute liver injury; acute kidney injury) occurred during hospitalization, were used in the model. A value of P < 0.05 was considered statistically significant.

## Results

### Clinical features of leptospirosis-infected patients with different clusters

**Figure 1:**
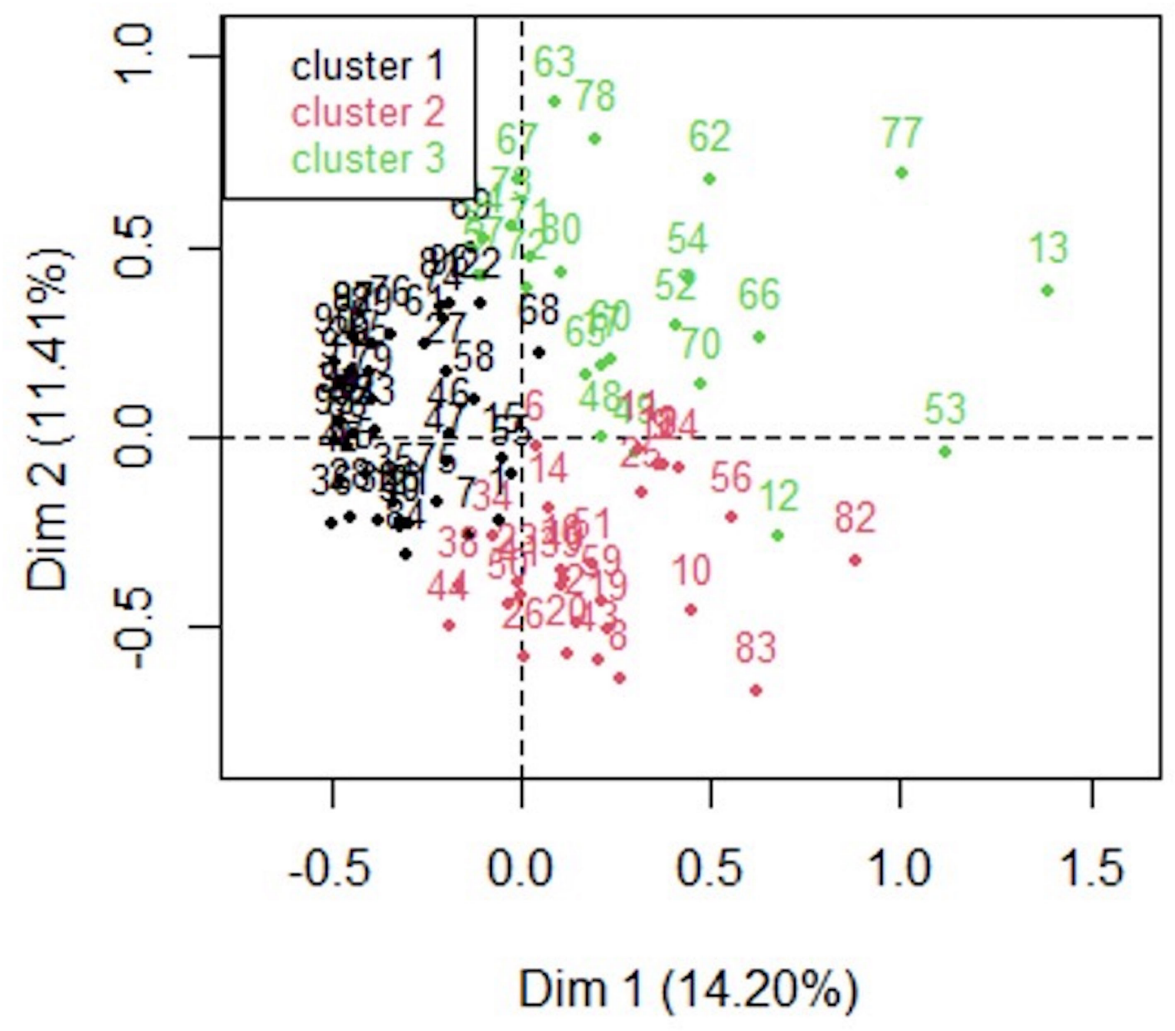
Map of clinical features of different leptospirosis. Two−dimensional distribution of clinical features of leptospirosis mapped along the two dimensions (dim 1 and dim 2) identified by multiple correspondence analysis. The relative positions of the patients in the plane are represented by different colors reflecting the subtypes distinguished by cluster analysis. Cluster 1 (black; n = 43) was the mild leptospirosis, cluster 2 (red; n = 28) was the respiratory presentation, cluster 3 (green; n = 25) was a hepato−renal form.

A total of 95 patients with leptospirosis from 9 tertiary care hospitals in Hunan China, were enrolled in the study. Patients were classified into 3 clusters based on clinical features with MCA analysis (**Figure 1**). The clinical features of patients from the 3 categories were compared and presented in **Table 1**. Cluster 1 (n=43, 45.3%) was the most prevalent clinical phenotype, with the lowest prevalence of organ dysfunction and shortest hospital stay. Cluster 2 (n=28, 29.5%) was characterized by symptoms including dyspnea, coughing, hemoptysis, and respiratory and circulatory failure. Cluster 3 (n=24, 25.3%) was distinguished by acute liver and renal failure in contrast with a low incidence of respiratory and circulatory failure. The difference in age distribution across the groups was not statistically significant (*p*=0.096), but the percentage of those above 65 was significant higher in cluster 3 of hepato-renal leptospirosis (41.7%), followed by cluster 2 of respiratory leptospirosis (21.4%) and cluster 1 (9.3%, *p*=0.008). Males constitute a substantially greater proportion of the incidence population than females, but there is no statistically significant difference in the distribution of males and females within each group. Of the 95 patients enrolled for this study, 50 (52.6%) were smokers and 45 (47.4%) were non-smokers, with a significant difference in the distribution between the three groups (*p*=0.028). At admission, fatigue was the most frequently reported symptoms (n=89, 93.7%), followed by fever (n=87, 91.6%), myalgia of the lower limbs (n=76, 80.0%), cough (n=53, 55.8%), hemoptysis (n=46, 48.4%), dyspnea (n=45, 47.4%), and expectoration (n=34, 35.8%). Myalgia of the lower limbs, altered consciousness, central nervous system symptoms, jaundice, diarrhea, abdominal pain, dyspnea, cough, hemoptysis, expectoration, and chest pain were distributed differently across the three groups of patients.

**Table 1:** Clinical manifestations of different clusters.

	Total (n=95)	Cluster 1 Mild leptospirosis (n=43)	Cluster 2 Respiratory leptospirosis (n=28)	Cluster 3 Hepato-renal leptospirosis (n=24)	*p* Value
Age, median (IQR), years	54.0 (39.0-65.0)	49.0 (35.0-61.0)	59,5 (38.2-65.0)	57.0 (44.0-68.7)	0.096
Age>65 years	20 (21.1%)	4 (9.3%)	6 (21.4%)	10 (41.7%)	0.008
Male, n [%]	82 (86.3%)	35 (81.4%)	24 (85.7%)	23 (95.8%)	0.273
Female, n[%]	13 (13.7%)	8 (18.6%)	4 (14.3%)	1 (4.2%)	
Smoking, n[%]	50 (52.6%)	25 (58.1%)	9 (32.1%)	16 (66.7%)	0.028
**Symptoms and signs, n[%]**					
Fever	87 (91.6%)	41 (95.3%)	25 (89.3%)	21 (87.5%)	0.404
Fatigue	89 (93.7%)	40 (93.0%)	25 (89.3%)	24 (100.0%)	0.363
Myalgia of lower limbs	76 (80.0%)	35 (81.4%)	18 (64.3%)	23 (95.8%)	0.018
Arthralgia	6 (6.3%)	1 (2.3%)	1 (3.6%)	4 (16.7%)	0.063
Altered consciousness	19 (20.0%)	1 (2.3%)	13 (46.4%)	5 (20.8%)	<0.001
Headache	21 (22.1%)	9 (20.9%)	4 (14.3%)	8 (33.3%)	0.248
CNS symptoms	25 (26.3%)	6 (14.0%)	11 (39.3%)	8 (33.3%)	0.040
Jaundice	26 (27.4%)	5 (11.6%)	2 (7.1%)	19 (79.2%)	<0.001
Vomiting	16 (16.8%)	7 (16.3%)	3 (10.7%)	6 (25.0%)	0.389
Diarrhea	10 (10.5%)	1 (2.3%)	2 (7.1%)	7 (29.2%)	0.004
Abdominal pain	11 (11.6%)	1 (2.3%)	3 (10.7%)	7 (29.2%)	0.004
Dyspnea	45 (47.4%)	6 (14.0%)	27 (96.4%)	12 (50.0%)	<0.001
Cough	53 (55.8%)	22 (51.2%)	21 (75.0%)	10 (41.7%)	0.039
Hemoptysis	46 (48.4%)	22 (51.2%)	19 (67.9%)	5 (20.8%)	0.003
Expectoration	34 (35.8%)	11 (25.6%)	18 (64.3%)	5 (20.8%)	0.001
Chest pain	9 (9.5%)	0 (0.0%)	3 (10.7%)	6 (25.0%)	0.002
Other bleeding	16 (16.8%)	4 (9.3%)	7 (25.0%)	5 (20.8%)	0.177
Gastrointestinal bleeding	10 (10.5%)	4 (9.3%)	4 (14.3%)	2 (8.3%)	
Epistaxis	3 (3.2%)	0 (0.0%)	2 (7.1%)	1 (4.2%)	
Haematuria	5 (5.3%)	0 (0.0%)	3 (10.7%)	2 (8.3%)	
Purpura	3 (3.2%)	0 (0.0%)	1 (3.6%)	2 (8.3%)	
Hepatomegaly	3 (3.2%)	0 (0.0%)	1 (3.6%)	2 (8.3%)	0.094
Splenomegaly	3 (3.2%)	0 (0.0%)	1 (3.6%)	2 (8.3%)	0.094
**Comorbidities, n[%]**					
Hypertension	11 (11.6%)	5 (11.6%)	2 (7.1%)	4 (16.7%)	0.598
Diabetes	5 (5.3%)	2 (4.7%)	1 (3.6%)	2 (8.3%)	0.715
Viral hepatitis	2 (2.1%)	1 (2.3%)	0 (0.0%)	1 (4.2%)	0.730
Anemia	4 (4.2%)	0 (0.0%)	2 (7.1%)	2 (8.3%)	0.112

Data are presented as medians (IQR) and n (%). P values were calculated by Kruskal-Wallis H test, Chi-square test or Fisher’s exact test, as appropriate. P values indicate differences among different clusters. IQR, interquartile range; CNS symptoms, central nervous system symptoms.

### Laboratory findings across the three clusters of patients

The laboratory findings were summarized in **Table 2**. The distribution of leukocytes, platelets, hemoglobin, neutrophil ratio, and lymphocyte ratio in routine blood tests varied significantly among the three groups of patients. In comparison to cluster 1 and cluster 2, the cluster 3 group had significantly more white blood cells (12.5[10.6-17.3 vs. 9.8[6.7-12.1] vs. 11.1[9.7-12.3], *p*=0.017), but significantly lower platelets (52.5[37.8-100.0] vs. 109.0[65.5-223.0] vs. 93.0[48.0-168.0], *p*=0.009) than other groups. In comparison to cluster 1, hemoglobin levels were significantly lower in the cluster 2 group, but not in the other groups. Additionally, the distribution of myocardial injury mediators was also considerably varied between the three groups of patients. Cluster 3 had a lower median albumin (27.5[24.2-30.1] vs. 34.5[30.6-37.7] vs 31.3[29.4-33.0], *p*<0.001), and higher levels of total (87.0[38.0-205.0] vs. 15.3[10.7-21.8] vs. 19.5[12.6-38.2], *p*<0.001) and direct bilirubin (53.0[20.1-156.0] vs. 6.1[4.4-11.2] vs. 9.6[5.2-19.5], *p*<0.001), which was in accordance with the classification methods in the article. Cluster 3 patients exhibited higher levels of renal dysfunction indicators such as blood urea nitrogen (22.6[11.3-25.6] vs. 7.8[5.0-10.4] vs. 11.3[8.3-14.8], *p*<0.001), creatinine (276.0[141.0-453.0] vs. 76.0[66.3-119.0] vs. 120.0[98.4-174.0], *p*<0.001) and uric acid (400.0[296.0-561.0] vs. 252.0[175.0-299.0] vs. 262.0[199,0-413.0], *p*=0.004) than the individuals in the other clusters. Creatine kinase and creatine kinase isoenzymes were significantly higher in cluster 2 patients than those in cluster 1 and cluster 3. Patients in cluster 3 exhibited significantly longer prothrombin time, activated partial thrombin time, and higher fibrinogen level than patients in the other clusters, with no statistically significant differences in thrombin time, d-dimer, globulin, alanine aminotransferase, aspartate aminotransferase and inflammatory mediators across the three groups.

**Table 2:** Laboratory findings of different clusters.

	Total (n=95)	Cluster 1 Mild leptospirosis (n=43)	Cluster 2 Respiratory leptospirosis (n=28)	Cluster 3 Hepato-renal leptospirosis (n=24)	*p* Value
**Blood Routine**					
White blood cell count, ×10^9^/L	11.1 (7.8-12.9)	9.8 (6.7-12.1)	11.1 (9.7-12.3)	12.5 (10.6-17.3)	0.017
Platelet count, ×10^9^/L	90.0 (46.0-168.0)	109.0 (65.5-223.0)	93.0 (48.0-168.0)	52.5 (37.8-100.0)	0.009
Hemoglobin, g/L	104.0 (85.3-122.0)	114.0 (93.8-125.0)	89.0 (78.5-118.0)	101.0 (82.5-118.0)	0.037
Neutrophil count, ×10^9^/L	9.4 (6.8-11.4)	7.7 (5.2-11.1)	9.7 (8.7-10.9)	10.4 (8.4-15.1)	0.102
Neutrophil percentage, %	87.2 (78.9-91.7)	82.2 (66.6-91.3)	88.3 (84.6-92.4)	88.0 (84.2-90.7)	0.026
Lymphocyte count, ×10^9^/L	0.8 (0.4-1.4)	1.0 (0.5-1.5)	0.7 (0.4-1.1)	0.7 (0.4-1.1)	0.323
Lymphocyte percentage, %	6.7 (4.1-14.7)	10.9 (4.7-22.9)	5.8 (3.7-10.0)	5.6 (3.4-11.0)	0.040
Eosinophil count, ×10^9^/L	0.0 (0.0-0.1)	0.0 (0.0-0.2)	0.0 (0.0-0.1)	0.0 (0.0-0.1)	0.061
Eosinophil percentage, %	0.1 (0-0.6)	0.1 (0.0-1.7)	0.01 (0.0-0.1)	0.120 (0.0-0.525)	0.134
**Blood Biochemistry**					
Albumin, g/L	31.0 (27.9-34.9)	34.5 (30.6-37.7)	31.1 (29.4-33.0)	27.5 (24.2-30.1)	<0.001
Globulin, g/L	25.8 (21.0-29.4)	25.8 (22.5-29.6)	24.9 (18.8-28.8)	27.5 (22.1-29.4)	0.432
Total bilirubin, μmol/L	20.8 (13.0-55.7)	15.3 (10.7-21.8)	19.5 (12.6-38.2)	87.0 (38.0-205.0)	<0.001
Direct bilirubin, μmol/L	10.6 (5.0-33.1)	6.1 (4.4-11.2)	9.6 (5.2-19.5)	53.0 (20.1-156.0)	<0.001
Alanine aminotransferase, U/L	50.9 (33.5-87.4)	49.7 (33.8-73.0)	45.6 (29.9-87.4)	55.5 (38.0-107.0)	0.435
Aspartate aminotransferase, U/L	53.0 (32.6-103.0)	44.0 (24.9-68.0)	57.0 (41.2-118.0)	58.6 (37.7-212.0)	0.068
Lactate dehydrogenase, U/L	311.0 (249.0-453.0)	287.0 (231.0-388.0)	391.0 (283.0-567.0)	346.0 (279.0-480.0)	0.047
Blood urea nitrogen, mmol/L	10.2 (6.4-15.2)	7.8 (5.0-10.4)	11.3 (8.3-14.8)	22.6 (11.3-25.6)	<0.001
Creatinine, μmol/L	116.0 (70.0-214.0)	76.0 (66.3-119.0)	120.0 (98.4-174.0)	276.0 (141.0-453.0)	<0.001
Uric acid, μmol/L	267.0 (191.0-395.0)	252.0 (175.0-299.0)	262.0 199.0-413.0)	400.0 (296.0-561.0)	0.004
**Myocardial Injury Mediators**					
Creatine kinase, U/L	246.0 (95.0-919.0)	118.0 (55.9-456.0)	535.0 (231.0-1060.0)	443.0 (159.0-2030.0)	0.010
Creatine kinase isoenzyme, U/L	23.0 (17.6-38.7)	18.4 (13.0-34.0)	28.0 (20.0-44.0)	24.1 (20.9-54.8)	0.031
Myoglobin, g/L	177.0 (86.0-604.0)	96.0 (46.9-281.0)	283.0 (136.0-785.0)	409.0 (165.0-868.0)	0.004
**Blood Coagulation**					
Prothrombin time, s	13.6 (12.5-14.8)	13.0 (12.1-14.0)	13.3 (12.5-14.7)	14.5 (13.7-16.0)	0.017
Activated partial thrombin time, s	40.0 (30.6-47.5)	33.4 (28.7-42.0)	38.3 (33.4-46.3)	44.8 (33.3-48.9)	0.044
Thrombin time, s	16.3 (14.8-17.2)	17.0 (16.0-17.9)	15.4 (14.1-16.8)	16.0 (13.9-16.8)	0.058
Fibrinogen, g/L	5.2 (4.2-6.6)	4.9 (3.2-6.0)	5.1 (4.3-5.7)	6.3 (5.2-7.8)	0.012
D-dimer, μg/mL	1.7(0.8-2.5)	1.2 (0.6-2.1)	2.3 (1.1-3.2)	1.6 (0.8-2.8)	0.171
**Inflammatory Mediators**					
C-reactive protein, mg/L	114.0 (70.2-178.0)	89.0 (51.1-157.0)	114.0 (98.6-146.0)	180.0 (93.6-216.0)	0.063
Procalcitonin, µg/L	4.0 (1.2-23.0)	2.1 (0.6-8.3)	16.4 (1.3-49.1)	6.2 (2.9-21.0)	0.055
ESR, mm/h	56.5 (34.0-82.3)	48.5 (36.5-74.5)	57.0 (24.0-87.5)	67.5 (40.5-84.8)	0.642

Data are presented as medians (IQR). P values were calculated by Kruskal-Wallis H test as appropriate. P values indicate differences among different clusters. IQR, interquartile range, ESR, Erythrocyte sedimentation rate.

### Organ dysfunction and outcomes of different clusters

We compared the organ dysfunction and outcomes across the various clusters in **Table 3**. Organ dysfunction occurred in the following proportion of all hospitalized leptospirosis patients: acute kidney injury (n=39, 41.1%), acute respiratory failure (n=38, 40.0%), acute liver injury (n=33, 35.1%), circulatory failure (n=31, 32.6%), multiple organ functional impairment (n=38, 40.0%), with significant differences between the three clusters. Specially, a *post-hoc* two-by-two assessment of significance levels using the Bonferroni method revealed that the incidence of acute respiratory failure and acute kidney injury was significantly different between any of two clusters. The incidence of acute liver injury was less common in cluster 1(7[16.3%] vs. 20[87.0%], *p*<0.001) and cluster 2(6[21.4%] vs. 20[87.0%], *p*<0.001) patients compared to cluster 3 individually. Multiple organ failure was more prevalent in cluster 2 (19[67.9%] vs. 4[9.3%] vs. 15[62.5%], *p*<0.001), resulting in more possibility to develop into severe leptospirosis requiring ICU admission (23[82.1%] vs. 5[11.6%] vs. 10[41.7%], *p*<0.001), more endotracheal intubation (17[60.7%] vs. 0[0.0%] vs. 5[20.8%], *p*<0.001) and longer ICU stays (8.0[1.0-10.7] vs. 0.0[0.0-0.0] vs. 0.0[0.0-3.5], *p*<0.001). In terms of prognosis, 80(84.2%) patients recovered from the disease while 15(15.8%) patients died. Individually, patients in cluster 2 (8[28.6%]) and cluster 3 (7[29.2%]) patients had a considerably greater mortality rate than those in cluster 1. However, no significant difference in mortality was observed between cluster 2 and cluster 3.

**Table 3:** Organ dysfunction and outcomes of different clusters.

	Total (n=95)	Cluster 1 Mild leptospirosis (n=43)	Cluster 2 Respiratory leptospirosis (n=28)	Cluster 3 Hepato-renal leptospirosis (n=24)	*p* Value
**Organ dysfunction, n[%]**					
Acute respiratory failure	38 (40.0%)	1 (2.3%)	28 (100%)	9 (37.5%)	<0.001
Circulatory failure	31 (32.6%)	7 (16.3%)	15 (53.6%)	9 (37.5%)	0.004
Acute liver injury	33 (35.1%)	7 (16.3%)	6 (21.4%)	20 (87.0%)	<0.001
Acute kidney injury	39 (41.1%)	5 (11.6%)	13 (46.4%)	21 (87.5%)	<0.001
Multiple organ failure	38 (40.0%0	4 (9.3%)	19 (67.9%)	15 (62.5%)	<0.001
**Clinical Outcomes**					
Endotracheal intubation, n[%]	22 (23.2%)	0 (0.0%)	17 (60.7%)	5 (20.8%)	<0.001
ICU admission, n[%]	38 (40.0%)	5 (11.6%)	23 (82.1%)	10 (41.7%)	<0.001
ICU stay length, median (IQR), days	0.0 (0.0-5.0)	0.0 (0.0-0.0)	8.0 (1.0-10.7)	0.0 (0.0-3.5)	<0.001
Total hospital stay length, median (IQR), days	9.0 (7.0-14.0)	9.0 (7.0-12.2)	10.0 (6.0-17.0)	11.0 (6.2-17.0)	0.773
Death, n[%]	15 (15.8%)	0 (0.0%)	8 (28.6%)	7 (29.2%)	<0.001

Data are presented as medians (IQR) and n (%). P values were calculated by Kruskal-Wallis H test, Chi-square test or Fisher’s exact test, as appropriate. P values indicate differences among different clusters. IQR, interquartile range; ICU, intensive care unit.

### Risk factors for ICU admission in severe leptospirosis patients

Univariate logistic regression analysis of risk factors for ICU admission in patients with severe leptospirosis revealed that dyspnea, altered consciousness, neutrophil count, lactate dehydrogenase, blood urea nitrogen, creatine kinase isoenzyme, activated partial thrombin time, d-dimer, c-reactive protein were significantly associated with ICU admission (**Table 4**). Several factors in univariate analysis plus age, gender and smoking situation were involved in the multivariate logistic regression analysis, which suggested that dyspnea (OR=19.051, 95%CI: 1.218-692.730, *p*=0.037) and neutrophil count (OR=1.611, 95%CI: 1.033-2.513, *p*=0.036) were risk factors for ICU admission. However, no promising predictors for mortality were identified in the current study (data not shown).

**Table 4:** Logistic regression analysis of risk factors for ICU admission in patients with severe leptospirosis.

Variables	Univariate analysis		Multivariate analysis			
	OR (95%CI)	*p* value	OR (95%CI)		*p* value	
Age	1.026 (1.000-1.053)	0.048	1.043 (0.968-1.124)	0.270	
Male	0.747 (0.230-2.423)	0.627	0.347 (0.007-18.282)	0.601	
Smoking	0.492 (0.214-1.132)	0.095	0.973 (0.086-11.012)	0.982	
**Clinical symptoms**					
Fever	0.642 (0.150-2.738)	0.549			
Fatigue	1.358 (0.236-7.813)	0.731			
Myalgia	0.525 (0.190-1.447)	0.213			
Jaundice	0.915 (0.363-2.307)	0.851			
Vomiting	0.634 (0.201-1.997)	0.436			
Dyspnea	6.588 (2.630-16.504)	<0.001	29.051 (1.218-692.730)	0.037	
Hemoptysis	1.891 (0.823-4.342)	0.133			
Altered consciousness	3.297 (1.159-9.380)	0.025	0.172 (0.008-3.874)	0.268	
**Laboratory findings on admission**					
White blood cell count, ×10^9^/L	1.077 (0.993-1.167)	0.072			
Platelet count, ×10^9^/L	0.996 (0.991-1.001)	0.082			
Neutrophil count	1.121 (1.016-1.238)	0.023	1.611 (1.033-2.513)	0.036	
Eosinophil count	0.100 (0.005-2.205)	0.144			
Albumin, g/L	0.933 (0.868-1.003)	0.060			
Total bilirubin, μmol/L	0.998 (0.995-1.002)	0.421			
Alanine aminotransferase, U/L	0.999 (0.997-1.002)	0.683			
Lactate dehydrogenase, U/L	1.005 (1.002-1.008)	0.003	1.004 (0.988-1.019)	0.631	
Blood urea nitrogen, mmol/L	1.059 (1.005-1.116)	0.033	1.066 (0.898-1.265)	0.465	
Creatinine, μmol/L	1.002 (0.999-1.005)	0.119			
Creatine kinase isoenzyme, U/L	1.025 (1.004-1.046)	0.022	1.010 (0.964-1.059)	0.672	
Activated partial thrombin time, s	1.046 (1.003-1.092)	0.036	1.060 (0.922-1.218)	0.414	
D-dimer, μg/mL	1.701 (1.044-2.773)	0.033	0.686 (0.257-1.832)	0.452	
C-reactive protein, mg/L	1.010 (1.002-1.018)	0.009	1.005 (0.991-1.019)	0.519	

OR, odds ratio; CI, confidence interval.

## Discussion

Leptospirosis is a zoonosis of protean manifestations (Peter, 1982) and can be easily misdiagnosed. Our study found a significantly higher incidence of leptospirosis in male than in female, which was consistent with the results of most previous studies (Kawaguchi et al., 2008). This might be related not only to occupation and exposure (Alvarado-Esquivel et al., 2015; Lau et al., 2016), but also to gender-based genetic and immune susceptibility (Puca et al., 2018). The age of onset was concentrated around the age of 50, which might be associated with more frequent exposure to risk factors for leptospirosis such as farming and fishing in this age group (Puca et al., 2018). These demographic features were less helpful for diagnosis.

Furthermore, the symptoms spectrum of leptospirosis is extremely broad, and there is no good clinical method to differentiate patients with leptospirosis by demographics, clinical symptoms, laboratory testing, and prognosis. In the current study, we conducted a MCA analysis based on clinical features, laboratory findings and outcomes, and identified three subtypes of populations with differences in ICU admission and mortality rate, which could be effective indicators for prognosis of patients with leptospirosis. Traditionally, distinct clinical syndromes of leptospirosis have been considered to be linked to specific serogroups. However, this view was questioned by some authorities (Edwards and Domm, 1960; Alexander et al., 1963; Feigin and Anderson, 1975), and more intense study over the past 50 years has refuted this hypothesis. In humans, severe leptospirosis is frequently but not invariably caused by serovars of the icterohaemorrhagiae serogroup. The infection of the specific serovars largely depend on the geographic location and the ecology of local maintenance hosts.

We identified three clusters: mild leptospirosis (cluster 1), respiratory leptospirosis (cluster 2), and hepato-renal leptospirosis (cluster 3).The current study did not identify a subtype of neurological leptospirosis, while, notably, we discovered that respiratory and hepato-renal leptospirosis had a higher prevalence of altered awareness and CNS symptoms than mild leptospirosis. Therefore, we speculated that neuro-leptospirosis was implicated in the kind of respiratory and hepato-renal leptospirosis due to the low incidence of neuro-leptospirosis, and the neurological symptoms were concealed by pulmonary or digestive symptoms, and the limitation of statistical approach. Studies suggested that neurological symptoms occurred in 10-15% of cases and were often neglected, so we should consider leptospirosis-associated neurological symptoms in areas where leptospirosis is endemic (Puca et al., 2017; Nabity et al., 2020). Mild leptospirosis of Cluster 1, which accounted for 45.3% of our patients, had the best prognosis among three clusters in this study, which was highly accordance with anicteric leptospirosis. The anicteric leptospirosis commonly begins abruptly and frequently presents with vague symptoms such as headache, myalgia (particularly in the calf muscles), ocular abnormalities, fever, nausea, vomiting, rash and epistaxis. Puca E et al. also showed that ocular and cutaneous involvement of leptospirosis usually did not cause serious complications and death (Puca et al., 2016). Cluster 2, mainly characterized by respiratory failure, had the high rate of endotracheal intubation that required ICU admission, was comparable with a pattern previously described in severe leptospirosis (Gouveia et al., 2008; Herath et al., 2019). The incidence of respiratory leptospirosis was about 10% in Brazil and French (Gouveia et al., 2008; Miailhe et al., 2019), compared to 29.5% in our population. Cluster 3 was distinguished by acute liver and kidney failure in contrast with slightly lower incidence of respiratory and circulatory failure. Second, as a temperate zone, there were still some outbreaks and a high incidence of leptospirosis in Hunan. Leptospirosis is a neglected zoonosis that causes considerable public health concerns throughout the world, particularly in Southeast Asian countries such as China, Indonesia, Vietnam, Malaysia, Cambodia and South Korea (Zhang et al., 2012; Loan et al., 2015; Zhang et al., 2019). It was discovered that leptospirosis morbidity rates varied across China’s provinces, with the warm and moist areas of the southern and central China being the predominant regions of leptospirosis infection (Zhang et al., 2020). Frequent rainfall can flush leptospirosis-contaminated soil into bodies of water, increasing the risk of human exposure to leptospirosis.

Notably, this study revealed that 40% of hospitalized leptospirosis cases in a temperate zone was adequately severe to transfer to ICU ward, 23.2% of patients required endotracheal intubation and longer ICU stays. The death rate for icteric leptospirosis has been reported to be as high as 5–15% (Levett, 2001) and 30–70% for leptospirosis-associated pulmonary hemorrhage syndrome (Dolhnikoff et al., 2007; Gouveia et al., 2008), compared to 20.8% and 60.7%, respectively, in our population. Leptospira interrogans serogroup Icterohaemorrh agiae serovar Lai (Hu et al., 2014) was the most common pathogen in Chinese leptospirosis patients, which was also the causative pathogen in 75% of the pulmonary diffuse hemorrhagic leptospirosis. The 15.8% mortality rate was slightly higher than the mean case fatality ratio of 6.85% (Costa et al., 2015), and the majority of leptospirosis deaths was due to renal failure and/or gastrointestinal, pulmonary, or cerebral hemorrhage (Vijayachari et al., 2008; Dong and Chen, 2021) caused by extensive capillary damage including extensive renal necrosis and glomerular atrophy. Moreover, leptospira increased the permeability and engorgement of blood vascular cells (Narayanavari et al., 2015; Matsunaga and Haake, 2018; Zavala-Alvarado et al., 2020). Given the prevalence and predominance of L. interrogans in China, the mortality rate was significantly higher than in other countries.

In this study, 11 patients were diagnosed using metagenomic next-generation sequencing (mNGS) of clinical samples including blood or alveolar lavage fluid. Several diagnostic methods have been used to confirm leptospirosis infection including pathogen culture, pathogen-specific antibodies detection, and genetic way such as mNGS. However, several limitations exist for above testing, for example: (1) Leptospirosis culture is less helpful and not commonly used for clinical diagnosis since it is technically difficult to culture due to the demand of fastidious culture media and long incubation time (Rodriguez et al., 2014); (2) The traditional antibody test of MAT is the most commonly used method to confirm leptospiraosis infection (Sykes et al., 2011), while identifying infecting serovar is difficult based on the cross-reactive MAT tests due to laboratory variation and differences in host-specific humoral immune responses (Markovich et al., 2012); (3) Several ELISAs for detecting IgM, IgG, or both antibodies have been developed to detect specific antibodies in leptospirosis, however, it has not been priorly recommended due to lower sensitivity compared to MAT test; (4) Genetic identification, such as mNGS, have become widely available for the diagnosis of leptospirosis although it is relatively expensive than traditional methods (Harkin et al., 2003). However, false-negative results can be avoided when facing low bacterial loads due to disease phase, complicated immune response, or administration of antimicrobial drugs (Levett et al., 2005; Waggoner and Pinsky, 2016). Thus, to some extends, mNGS, as a method allowing for high-throughputand massively parallel sequencing, benefits leptospirosis patients a lot, particular for those patients with suspicious symptoms, negative traditional testing, or emergent conditions, thus avoiding inadequate diagnosis, delayed treatment, and increased morbidity and mortality. Over the past five years, mNGS testing has been widely used for clinical diagnosis of microbiology examinations (Simner et al., 2018) due to the advantages of quicker diagnosis, higher sensitivity, and even detecting pathogen subtypes as well. Therefore, to some extends, mNGS can avoid some disadvantages of traditional testing, and represent as a promising supplementary tool for some uncommon pathogen diagnosis.

In the current study, we identified several possible predictors, such as dyspnea and neutrophil count, for ICU admission in hospitalized leptospirosis. However, no significant predictors were determined to be associated with mortality of leptospirosis in the current investigation, which might be due to the small sample size. Age has already been shown as a significant predictor of leptospirosis mortality (Chang et al., 2005). Oliguria, hypotension and abnormal chest auscultation were all found to be independent predictors of severe leptospirosis mortality in a Polynesian cohort (Doudier et al., 2006). According to a French study, the following variables including dyspnea, oliguria, increased white blood cell count, repolarization abnormalities on electrocardiograms and alveolar infiltrates on chest radiographs were reported to be associated with leptospirosis mortality (Dupont et al., 1997). Additionally, a Thai cohort demonstrated that hypotension, oliguria, hyperkalaemia, and presence of pulmonary rales were linked to the mortality of leptospirosis. While oliguria has been frequently shown to be related to mortality in numerous earlier studies, our findings are different, and we believe they are justified. Patients in the current study were admitted to tertiary care referral centers with fully functional haemodialysis units, and the patients with AKI were dialyzed whenever indicated. This, we believe, is the cornerstone of leptospirosis care and is likely to have resulted in a decrease in AKI mortality.

### Strengths and limitations

This study was limited by a few factors that should be considered. First, this was a retrospective study in which a relatively small subset of leptospirosis patients from Hunan. Second, selection bias may have been introduced since female were less likely to be included, and a large cohort study would be warranted to further strengthen our findings. Thirdly, limited cases of neurological leptospirosis were included in the study; thus, the conclusions may not apply to patients with neuro-leptospirosis.

## Conclusions

In the current study, we identified three phenotypes of leptospirosis with distinct clinical features and prognosis, which would assist clinicians in recognizing leptospirosis-like illness while also providing prognostic predictors. Additionally, dyspnea and neutrophil count were found to be independent risk factors for severe leptospirosis progression. When patients are infected with leptospira, it is prudent to consider more rigorous surveillance and therapy.

## Data availability statement

The original contributions presented in the study are included in the article/supplementary material. Further inquiries can be directed to the corresponding authors.

## Ethics statement

The studies involving human participants were reviewed and approved by The institutional review boards (IRBs) in Xiangya Hospital of Central South University (No. 202104005). Written informed consent for participation was not required for this study in accordance with the national legislation and the institutional requirements.

## Author contributions

All authors contributed to the study conception and design. Material preparation was performed by DL, HL. Data collection and analysis were performed by DL, HL and other authors. The first draft of the manuscript was written by DL and HL. Supervision was performed by PP and YAZ. All authors contributed to the article and approved the submitted version.

## Funding

This work was supported by Natural Science Foundation of China (No.82100037, No.81770080), National Key R&D Program of China (No. 2016YFC1304204), National Science Foundation for Post-doctoral Scientists of China (No. 2021TQ0375), Hunan Outstanding Postdoctoral Innovative Talents Program (No.2021RC2018), Key R&D Prpgram of Hunan Province (No.2022SK2038) and Youth Foundation of Xiangya Hospital (No.2020Q06).

## Conflict of interest

The authors declare that the research was conducted in the absence of any commercial or financial relationships that could be construed as a potential conflict of interest.

## Publisher’s note

All claims expressed in this article are solely those of the authors and do not necessarily represent those of their affiliated organizations, or those of the publisher, the editors and the reviewers. Any product that may be evaluated in this article, or claim that may be made by its manufacturer, is not guaranteed or endorsed by the publisher.
